# Carbon Neutrality in the Middle East and North Africa: The Roles of Renewable Energy, Economic Growth, and Government Effectiveness

**DOI:** 10.3390/ijerph191710676

**Published:** 2022-08-27

**Authors:** Chuimin Kong, Jijian Zhang, Albert Henry Ntarmah, Yusheng Kong, Hong Zhao

**Affiliations:** School of Finance and Economics, Jiangsu University, Zhenjiang 212013, China

**Keywords:** carbon neutrality, renewable energy, economic growth, government effectiveness, MENA region

## Abstract

Carbon neutrality is a 21st-century priority area, with the Middle East and North Africa (MENA) countries making significant investments in renewable energy and climate mitigation initiatives to attain it. However, carbon neutrality research in the MENA region is under-developed, particularly when considering the roles of renewable energy, economic growth, and effectiveness of government. To address this gap, this research investigates the roles of renewable energy, economic growth, and government effectiveness toward the MENA region’s carbon neutrality goal. We implemented heterogeneous and second-generation panel data techniques that are resilient to cross-sectional dependency and slope heterogeneity to panel data spanning 16 MENA countries from 1996 to 2018. We discovered that MENA data are cross-sectionally dependent, heterogeneous, and cointegrated. We found that government effectiveness and renewable energy bring carbon neutrality closer, but economic growth initially delays it. We detected Environmental Kuznets Curve (EKC) in the MENA region, specifically in the High-Income Countries. Although there were signs of EKC in the Middle-Income Countries, this was not significantly validated. Finally, we found a one-way causal link from government effectiveness and renewable energy to carbon neutrality but a feedback mechanism between economic growth and carbon neutrality in the MENA region. As a result of these findings, it is recommended that the MENA region’s policymakers prioritize renewable energies and improve the effectiveness of government to drive economic growth toward the carbon neutrality goal.

## 1. Introduction

At the United Nations Climate Change Conference in 2015, an agreement (the Paris Agreement) was reached to move towards carbon neutrality (CN) by the end of the 21st century to reduce the consequences of climate change. CN refers to achieving “net-zero emission” of greenhouse gases (GHG) by balancing the created carbon emissions (CO_2_) utilizing carbon capture, storage, and conversion within a specific period. The Intergovernmental Panel on Climate Change (IPCC) special report “Global Warming of 1.5 °C” released in 2018, explicitly explains the importance of CN targets [1]. Over the last 15 years, CN has evolved from a hazy scientific concept to a key subject in the climate-change discussion. The United Kingdom became the first country to legislate for GHG reductions with the passage of the Climate Change Act in 2008 [2]. Following that, the Paris Agreement’s 1.5 °C climate objective asks for global “net-zero emissions” by 2050. The Paris Agreement bridges today’s policies to CN by the end of the century. After the Paris Climate Conference (COP21), several governments began to move toward the CN goal. At the moment, 126 governments and organizations throughout the world (particularly those vulnerable to climate actions) have pledged to attain CN.

The Middle East and North Africa (MENA) area is one of the most vulnerable to climate change, with extreme temperatures, desertification, coastal ecosystems, and high levels of air pollution [3]. Due to this, the MENA region is set to take the lead in climate mitigation and adaptation activities. Recently, Egypt joined five other nations to create the Adaptation Efforts Coalition, which aims to expedite global adaptation action to help achieve a climate-resilient society by 2030. Just before the COP26 summit in Glasgow, several MENA nations promised to reach net-zero emissions by 2050–2060. These countries also discussed their plans for achieving the CN objective, such as the Middle East Green Initiative and the framework for a carbon circular economy. With the next two COP meetings in the MENA region, COP27 (in Egypt) and COP28 (in the UAE), MENA countries are in a unique position to demonstrate climate policy leadership and advance the global climate-change management agenda by hosting successful back-to-back conferences that push world nations to achieve a carbon-neutral world [4].

The MENA region’s governments have committed to achieving the Paris accord and, as such, are transitioning their energy systems away from hydrocarbons and toward low-carbon technology [1]. A green, resilient, and inclusive approach to development can usher in a new model of growth for the MENA, creating jobs while providing the benefits of climate resilience, decarbonization, and cleaner air and water [3,4,5]. To reap the benefits of these investments, MENA will need to prioritize important transformation areas. The MENA Roadmap for Climate Action integrates climate action with development, improves institutions, removes impediments to private sector participation, promotes regional integration, and stimulates the creation of resilient and inclusive communities [5].

However, the United Nations Environment Programme’s (UNEP) “Emissions Gap Report 2019” shows that there is still a significant gap between nations’ (including those in MENA) commitments to cut CO_2_ and the 1.5 °C objectives [1]. While most MENA states have committed to ambitious objectives in accordance with global expectations, it will be interesting to know how successful their governments are and how these commitments are implemented within the context of significant economic activity in the area. For example, how effectively is economic growth (EGR) connected with the CN target, such that growth contributes to climate actions in the MENA region. Although there is recent research on the topic, most CN research focused on China [6,7], the United States [8,9], and other developed countries, such as the United Kingdom and the European Union [6,10,11], with little emphasis on countries such as MENA countries. In addition, many environmental studies, including those in the MENA area, concentrated on growth–environmental results [12,13,14], finance–environmental outcomes [15,16], urbanization–growth–environmental outcomes [17,18,19], and energy–growth–environmental outcomes [20,21,22], with minimal attention to government effectiveness. As a result, renewable-energy shifting coupled with the region’s government’s role in CN is under-developed. Achieving CN requires key initiatives. For instance, switching from traditional energies (fossil fuels) to renewable energies, such as wind and solar power, with high-level government commitments could considerably contribute to the goal. CN in MENA remains a concept rather than a reality due to a lack of empirical study investigating the progress of MENA nations and specific policy suggestions to achieve the carbon-neutral target in the region.

As CN is widely pushed across the world, this research intends to investigate the roles of renewable energy (RE), government effectiveness (GEF), and EGR in achieving this goal in the MENA region. The study specifically investigates the RE–GEF–EGR–CN relationship in the MENA area. It also investigates CN within the Environmental Kuznets curve (EKC) framework. Finally, it sub-groups the MENA countries into high-income countries (HICs) and middle-income countries (MICs) to better assess the progress these heterogeneous groups are making toward the overall MENA CN goal.

The following are the paper’s novelties. Aside from the usual growth–emission-reduction studies, our study joins the recent CN research within the context of the MENA region where literature is under-developed. Therefore, focusing on MENA countries is a new way of widening empirical discussions on CN aimed at identifying the realistic nature of the concepts across the world. Second, this study empirically incorporates GEF and RE into growth–CN in the MENA region while controlling for NRE and URB. This helps to understand how RE shift from traditional energy sources combined with the effective government could contribute to CN in the region. Generally, the role of GEF is missing in environmental literature. Considering the ambitious target—CN in a climate-risk region such as MENA—the government is responsible for bringing together key stakeholders to develop quality and practicable CN policies and frameworks as well as strict commitments to the implementation of these policies. Therefore, the study tries to establish how GEF could contribute to CN (as measured in terms of consumption-based emissions) in the MENA region as a way of rethinking GEF in CN targets. Third, this study accounted for income heterogeneity by analyzing the topic from the whole sample (all selected MENA countries) and income levels—HICs and MICs. This provides an in-depth understanding and progress of CN across countries of different income levels. We envisage that countries’ income levels could be linked to the level of commitments and investments in CN. Therefore, it is important income levels are considered when studying this topic. Finally, this study combined second-generation panel econometric models, including Westerlund–Edgerton bootstrapping cointegration tests, Augmented Mean Group (AMG), and Common Correlated Effect Mean Group (CCEMG) estimators, and panel granger causality (proposed by [23]) tests to investigate the topic. The models are robust, reliable, and account for endogeneity problems in the estimation process.

The remainder of the paper is set out as follows: Section 2 will focus on the literature review; Section 3 will deal with methodology; while Section 4 will present the results and discussion; and, finally, Section 5 will present the conclusions.

## 2. Literature

Systems of CN strategies are increasingly being adopted to confront the danger of climate change. Within this framework, authors have used single-country and panel data approaches to examine pathways to CN. From a single country perspective, Abbasi et al. [24] investigated the impact of energy usage (EU), natural resource depletion (NRD), EGR, population growth (POP), and industrial value added (IVA) on the CN of the UK. With CO_2_ as an index of CN, the authors implemented a dynamic Autoregressive–Distributed–Lag (ARDL) model on time series data from 1970 to 2019. The study found that EGR and NRD prolong CN in the short term while EU and POP contribute to the CN agenda in the long term. Udemba and Alola [25] investigated the impact of possible shocks from Australia’s ‘Direct Action’ policy in RE, fossil fuel energy, and foreign direct investment (FDI) in CN. They used the country’s national data from 1996 Q1 to 2018 Q4 and multiple scientific methodologies—structural break test and short- and long-run asymmetric relationships—for effective research and discussion of the study’s conclusions. The findings revealed that EGR and FDI had a detrimental impact on Australia’s CN by increasing the country’s CO_2_.

Fragkos et al. [26] used a country-level method to examine the implications of a low-emission route for 2050 on emissions, economic, and energy systems. The study revealed that RE development, along with EU electrification and improvement in energy efficiency, are the significant low-emission pathways strategies of many of the aforementioned economies—Indonesia, the United States, China, Australia, the Republic of Korea, Brazil, Russia, Canada, Japan, and India. Furthermore, the analysis indicated that each country’s specificities, goals, and natural resources are the primary drivers that support low-emission technology breakthroughs for nuclear, carbon capture and storage (CCS), and advanced biofuels. Similarly, Munir and Riaz [27] investigated the short- and long-run environmental implications of asymmetric patterns in energy types (oil, gas, coal, and electricity use) in Australia, China, and the United States. Munir and Riaz [27] used the NARDL to demonstrate that rising oil and coal usage are the key drivers that exacerbate CO_2_ in Australia in the long term. Furthermore, rising oil, coal, and gas use worsens environmental deterioration in the long run in the United States, whereas oil, gas, and electricity are responsible for severe environmental degradation in China. However, a negative shock in Australia’s power, oil, and gas energy use is much desired for reducing CO_2_. While a shock in electricity, gas, and oil energy consumption reduces CO_2_ in China, a shock in solely coal, electricity, and gas consumption reduces CO_2_ significantly in the United States. Furthermore, Shahbaz et al. [28] and Rahman and Vu [17] are among the researchers focusing on the relationship between CO_2_ and energy, EGR, and non-EGR in Australia. Shahbaz et al. [28] discovered that Australia’s outstanding EGR is not emission-intensive, while the opposite is true for energy.

Although environmental discussions are ongoing in the MENA region, empirical discussions on CN to guide policy formulation towards the CN goal are under-represented. Awad and Abugamos [29] studied the income–CO_2_ Nexus for MENA nations using a semi-parametric technique on a panel of 20 MENA countries from 1980 to 2014. The study found an inverted-U link between income and CO_2_, lending credence to EKC in the MENA area. However, the authors failed to account for income disparities between the nations investigated. Sileem [30] used a modified EKC to analyze the growth–environmental deterioration in MENA nations from 2004 to 2013. Using a fixed-effects model, the empirical results show that EKC exists in the MENA region. The Granger causality test findings demonstrate the existence of a one-way link between CO_2_ and corruption. Andriamahery and Qamruzzaman [14] conducted a comparative analysis of the influence of RE, energy innovation, and trade openness on environmental sustainability in Tunisia and Morocco from 1980 to 2018. They employed linear ARDL, nonlinear ARDL, and Granger causality tests. The study discovered a long-term relationship between environmental sustainability, renewable energy, energy innovation, and trade openness for both nations. Furthermore, in terms of EKC, study findings using ARDL and nonlinear ARDL verified the EKC concept for Tunisia and Morocco. Finally, the direction causality test revealed unidirectional causation between renewable energy and environmental sustainability, as well as trade and environmental sustainability, but a bidirectional association between energy innovation and environmental sustainability. It is crucial to note that this study was undertaken on a country-by-country basis, therefore the findings and conclusions made may not apply to all MENA nations. All these authors concentrated on general environmental research without considering CN, which is critical in current environmental research.

Extending on the work of Apergis and Payne [31] and Payne [32], Arouri et al. [12] used bootstrap panel unit root tests and cointegration approaches to evaluate the link between CO_2_, EC, and EGR in 12 MENA nations from 1981 to 2005. Their findings reveal that in the long run, EC influences CO_2_ while EKC evidence was weak. Al-Rawashdeh, Jaradat, and Al-Shboul [33] evaluated the association between EGR and CO_2_ across the MENA region. The study showed no indication of EKC for MENA nations as a whole based on cointegration analysis of time series data from 1960 to 2010. However, EKC was discovered at the country level in Algeria, Tunisia, Yemen, Morocco, Turkey, and Libya. Similarly, Arouri et al. [12] examined the EKC theory empirically for 12 MENA countries from 1981 to 2005. The empirical findings of bootstrap panel unit root tests and cointegration approaches found evidence of EKC in the region’s most industrialized and diverse countries, but not in the region’s less industrialized countries.

In discussing CN literature, it is important to recognize the contributions of the numerous environmental research (see Table 1 for the summary of prior environmental literature). Although the findings are diverse, they still provided the basis for the current and future environmental discussions. Beyond this environmental literature, the focus now is the CN target. This has renewed the interest of policymakers and researchers across the world (see Table 2 for a summary of CN research). From Table 2, literature focusing on CN are new and mostly focused on the developed HICs, such as China, the USA, the UK, Australia, and the G-7 countries. With many countries pledging to attain CN, research in this area must not be limited to developed regions but should consider countries in the other parts (especially the climate-risk MENA region) of the world. Interestingly, Qin et al. [6] and Udemba and Alola [25] found RE promotes CN in China and Australia, while Hu et al. [34] revealed consumption of RE increases CO_2_ in India demonstrating divergent outcomes on a common initiative (RE shift) towards CN from different contexts.

It is clear from the literature that attempts have been made toward understanding environmental outcomes and their influencing factors. Nonetheless, as previously stated, the findings are conflicting. The literature on EGR, RE, and other environmental influences are substantial. However, emphasis on CN objectives is limited, particularly in the MENA region. Furthermore, empirical studies concentrating on CN while taking into account GEF, EGR, and RE are uncommon. It is clear from the literature that GEF in environmental research is overlooked. The effectiveness of government plays a role in achieving environmental outcomes. For instance, Pushak, Tiongson, and Varoudakis [53] discovered in their study that macroeconomic stability and public expenditure may have a greater growth payoff in nations with comparatively effective governments. Similarly, Gani [54] noted that nations with effective governments (low bureaucracy, efficient public service, and a focus on financial integrity and better management of public resources) may acquire producers’ trust and more effectively enforce governmental laws and regulations to reduce CO_2_ emissions. According to Yasmeen et al. [55], GEF has a role in lowering CO_2_ in both developed and developing nations since they are concerned with enhancing agricultural production efficiency. Considering this ambitious target—CN that requires continuous policy formulations and strict commitments towards this goal—the role of government cannot be ignored, especially in a climate-risk region such as MENA. Besides GEF, the role of EGR in CN is critical. Economic activity is frequently cited as the primary source of CO_2_. Indeed, EGR is critical to improving people’s lives. According to the EKC concept, individuals with higher incomes and more environmental knowledge will want lower environmental pollution in the long term. Lowering environmental pollution is helpful for both greater EGR and human quality of life. This is the EKC hypothesis’s primary environmental protection mechanism. The evidence for the EKC theory is found in several studies [16,56,57]. Furthermore, EGR produced by improved technology can permit larger output while emitting less CO_2_ [56,58]. Additionally, as the world strives to connect energy strategies with climate change such that countries are now becoming either foot-dragging or pace-setting [11], it is important to study RE within MENA region’s CN framework. To the best of our knowledge, our study is the first to combine RE, EGR, and GEF to examine CN in the MENA region while controlling for other emission determinants. Given the massive investment in RE sources and growth–emission mitigation initiatives in the MENA region, where climate action is prevalent, and with the majority of these countries pledging to achieve this goal, the role of government in committing to these pledges and implementing carbon neutral policies cannot be overlooked. As a result, this study investigates the roles of RE, EGR, and GEF in achieving CN in MENA countries.

## 3. Materials and Methods

### 3.1. Theoretical Rationale and Model Construction

Before setting out the econometric framework for exploring the roles of RE, GEF, and EGR in achieving the MENA region’s CN agenda, we provide a theoretical rationale guiding the selection of the variables of the study. CN, which is the outcome variable, is widely measured using CO_2_. CN is defined either based on CO_2_ only or on all greenhouse gases, however, in this study we chose the former which is consistent with literature [37,38,40,59], given that reduction in CO_2_ approaches CN agenda. According to the International Renewable Energy Agency [60], current trends of RE deployment in the MENA region show that the renewable-energy landscape is rapidly evolving and significant developments have taken place. In 2016, USD 11 billion was invested in renewables across the Arab region compared to USD 1.2 billion in 2008, or a nine-fold increase in only eight years [60]. In keeping with the MENA region’s energy deployment and the global need for alternative energy sources, we included RE in the CN framework, which is consistent with literature [16,17,40].

Given the far-reaching nature of the CN goal, especially in the MENA region, GEF in the form of “perceptions of the quality of public services, the quality of the civil service and its independence from political pressures, the quality of policy formulation and implementation, and the credibility of the government’s commitment to such policies” will play a role in achieving the goal [61]. Achieving CN within the context of human and economic activities would necessitate effective government that will constantly enhance the level of developing rules and regulations, market oversight, law enforcement, policy implementation, and government services [62]. The MENA region has taken governance initiatives, such as the MENA-OECD Initiative which began in 2003, as guarantees that continual policy initiatives are taken to promote public sector reform and to build and enhance institutions for good governance [63]. Therefore, we chose GEF as one of the determinants of CN in the MENA region.

EGR is selected for the study due to the theoretical proposition of EKC that put forward three channels (scale, composition, and technique effects) through which EGR affects CO_2_ [64]. According to the scale effect, EGR increases CO_2_ since increased production volumes necessitate more exploitation of natural resources and hazardous emissions. The composition impact suggests that EGR might reduce or worsen CO_2_ depending on economic structural changes. A structural transformation in the economy from agricultural to industrial activities increases CO_2_ as a country’s EGR rises, but a shift from energy-intensive industries to services and knowledge-based inventions and technology decreases CO_2_. Finally, the technique effect suggests that EGR reduces CO_2_ because higher income levels result in more investment in research and development (R&D), resulting in new and environmentally friendly technologies that replace old and highly polluting technologies, as well as strict environmental regulations and industry standards [65]. According to Jebli et al. [43] and Sarkodie et al. [46], empirical data corroborate the EKC theoretical stance.

Besides these selected variables, we need to control for other variables to improve statistical accuracy. The conversion of Earth’s land surface to urban use is harmful to the environment. Anthropogenic activities, such as the flight of a large number of vehicles, farming, and industrial operations, produce CO_2_ [66,67]. As a result, in this study, we control for urbanization (URB). Scholars, such as Khan et al. [59] and Xue et al. [19] endorse this theoretical justification for using URB in the transition to CN. CN is closely tied to the production and consumption of non-renewable energy (NRE), which increases CO_2_ [68,69]. Therefore, we control for NRE in CN framework.

Based on the theoretical arguments put forward, we proposed a function to estimate *CN* in a multivariate framework as:

(1) CN=f(GEF,NRE,EGR,URB,RE)

where *CN* is the outcome variable representing carbon neutrality; *GEF*, *NRE*, *EGR*, *URB*, and *RE* are the explanatory variables representing government effectiveness, non-renewable energy, economic growth, urbanization, and renewable energy, respectively. Equation (1) can be represented in panel data time series form as:

(2) $$CN_{it}=\alpha_{i}+\beta_{1}GEF_{it}+\beta_{2}NRE_{it}+\beta_{3}EGR_{it}+\beta_{4}URB_{it}+\beta_{5}RE_{it}+\mu_{it}$$

where $\beta_{1},\;\beta_{2},\beta_{3},\;\beta_{4},\;\;\mathrm{and}\;\beta_{5}$ are the coefficients of *GEF_it_*, *NRE_it_*, *EGR_it_*, *URB_it_*, and *RE_it_*, respectively. $\alpha_{i}\;\;\mathrm{and}\;\mu_{it}$ represent the time-invariant country-specific effect and the stochastic white noise error term, respectively. i denotes individual countries while t representing time-span. CN is measured in terms of CO_2_. As a normal practice in econometric analysis to reduce heteroscedasticity and capture elasticities, we transform the data into a natural log. We can therefore transform Equation (2) into log-linear while incorporating the specific CN measure (CO_2_) as:

(3) $$\begin{array}{l} \ln CO2_{it}=\alpha_{i}+\beta_{1}\ln GEF_{it}+\beta_{2}\ln NRE_{it}+\beta_{3}\ln EGR_{it}+\\ \;\;\;\;\;\;\;\;\;\;\;\;\;\;\;\;\;\;\;\;\;\;\beta_{4}\ln URB_{it}+\beta_{5}\ln RE_{it}+\mu_{it} \end{array}$$

For these variables to contribute to achieving CN, the elasticities $\beta_{1},\;\beta_{2},\beta_{3},\;\beta_{4},\;\;\mathrm{and}\;\beta_{5}$ must be significantly negative, otherwise, the variable is said to delay CN. Within the CN framework, the theoretical argument of EKC cannot be ignored. Therefore, we modify Equation (3) to capture the EKC model as:

(4) $$\begin{array}{l} \ln CO2_{it}=\alpha_{i}+\beta_{1}\ln GEF_{it}+\beta_{2}\ln NRE_{it}+\beta_{3}\ln EGR_{it}+\beta_{4}\ln EGR2_{it}+\\ \;\;\;\;\;\;\;\;\;\;\;\;\;\;\;\;\;\;\;\;\;\;\;\beta_{5}\ln URB_{it}+\beta_{6}\ln RE_{it}+\mu_{it} \end{array}$$

where ln *EGR*2 represents *EGR* in quadratic form. The coefficients must be significantly positive and negative in order for *EKC* to be verified.

### 3.2. Data Source

Since GEF data starts in 1996, this study used annual data of MENA countries for all the variables available from 1996–2018. All the countries without data on one or more variables (especially those with no investment records in RE) were excluded from the study. There were 16 MENA countries involved in the study. Following the literature [10,14,24], we measured CN in terms of CO_2_ (metric tons per capita) RE in terms of renewable-energy consumption (% of total final energy consumption), EGR in terms of GDP per capita (constant 2015 US$), NRE in terms total non-renewable-energy GEF in terms of the estimate of governance (ranges from approximately −2.5 (weak) to 2.5 (strong) governance performance), and URB in terms of urban population (% of the total population). The study sourced CO_2_, RE, NRE, URB, and EGR data from the World Bank (through World Development Indicators [66] and GEF data from the Worldwide Governance Indicators (WGI) databases). CO_2_ used in this study refers to carbon dioxide generated as a result of the consumption of solid, liquid, and gas fuels, as well as gas flaring.

### 3.3. Descriptive Statistics

The descriptive statistics of the variables selected for this study are presented in Table 3. Table 3 shows that there were 368 observations comprising 138 for HICs and 230 for MICs, suggesting more MICs countries (10 countries) were involved in the study than HICs (six countries). Generally, the mean values of the variables are higher in HICs than MICs indicating heterogeneity in MENA data. In terms of skewness, EGR, CO_2_, URB, NRE, and GEF are negatively skewed across the panels while RE is positively skewed. Concerning kurtosis, the results in Table 3 show that EGR in MENA, RE in HICs, and CO_2_, URB, and NRE in MICS exhibited normal distribution (kurtosis value equivalent to 3). However, the results show platykurtic distribution (kurtosis value less than 3) for (i) CO_2_, NRE, RE, and GEF in MENA as a whole; (ii) RE and GEF in MICs; and (iii) CO_2_, URB, NRE, and GEF in HICs. URB in MENA, EGR in HICs, and MICs exhibited leptokurtic distribution (kurtosis value greater than 3). The descriptive results generally show that the data is not normally distributed. This is further confirmed by the Jarque–Bera and probability results. Hence, heterogeneous panel data models will be appropriate for estimating the results [39].

The correlation findings in Table 4 indicate associations among explanatory variables and the outcome variable suggesting that the variables are linked. This provides an initial justification for studying the influence of the explanatory variables on the outcome variables. Furthermore, Table 4 presents the collinearity statistics to establish whether multicollinearity exists among the explanatory variables. The general rule is that a tolerance (Tol) value greater than 0.2 and a Variance Inflation Factor (VIF) value less than 5 indicate the absence of multi-collinearity. As depicted in Table 4, the Tol and VIF values show that the tolerance values are greater than 0.2 and VIF values are less than 5, signifying that multicollinearity is not a problem among the explanatory variables for the CO_2_ model.

### 3.4. Econometric Approaches

#### 3.4.1. Cross-Sectional Dependence (CD) and Slope Homogeneity Testing

Testing for CD and homogeneity among the variables provide a guide for selecting appropriate panel econometric tests for estimating the results. MENA countries are linked to one another in some way due to commerce and other socio-economic activity. Because of these close ties, there is a good chance that the data from these nations will show cross-sectional interdependence. According to Li Hu et al. [70], ignoring cross-sectional correlations may result in incorrect estimations and conclusions. Therefore, the study tested CD among the series Pesaran [71,72] CD tests to determine the existence or lack of dependencies in the panels. Secondly, since the ignorance of heterogeneity could lead to biased estimates and extrapolations [73], the researchers tested the heterogeneity assumption through the Pesaran–Yamagata [74].

#### 3.4.2. Panel Unit Roots Testing (PURT)

Following CD and slope homogeneity testing, PURT is performed. This is very important since it aids in the selection of a suitable estimator to estimate the results. It aids in determining the integrated order of the variables as a prerequisite for selecting a valid, reliable, and consistent estimator. The Cross-Sectional Augmented PURT (CIPS) and Pesaran [75] PURT in the Presence of Cross-Section Dependence (CADF) methods were employed in the study to check for unit roots in the data. The CIPS and CADF tests are widely accepted and proven to be reliable for testing unit roots in the presence of cross-sectional dependency and heterogeneous panels as revealed by this study [76,77].

#### 3.4.3. Panel Cointegration Testing

The next step after testing unit roots is checking for the existence or nonexistence of cointegration amid the variables. In the presence of cross-sectional dependence and unit roots, the Westerlund [78] cointegration test has proven to be valid and reliable in testing for cointegration among the variables [79]. The test is one of the most popular and preferred cointegration tests, especially where cross-sectional dependence is present among the variables. The Westerlund [78] test involves four-panel cointegration tests-intergroup (Gt), intergroup (Ga), inter-panel (Pt), and inter-panel (Pa) tests that are based on structural dynamics rather than residuals and, thus, do not include general limiting factors [16]. Furthermore, the Durbin-Hausman Panel Cointegration test developed by Westerlund [74] is utilized to assess cointegration. This test employs two distinct tests: the Durbin–Hausman panel (DHp) and the Durbin–Hausman group (DHg) [80]. Both Westerlund’s [78,80] tests were considered appropriate due to their suitability in handling CD and slope heterogeneity and have proven to be reliable in panel cointegration estimation [44,81].

#### 3.4.4. Panel Model Estimations

We implemented panel data estimators that are resilient to cross-sectional dependency, slope heterogeneity, unit roots, and cointegration in the data to produce valid and accurate estimates in the presence of CD, cointegration, and heterogeneity. As a result, in this study, the CCEMG and AMG estimators were utilized.

Pesaran [82] devised the CCEMG, which Kapetanios et al. [83] enhanced. The CCEMG estimator is based on the Common Correlated Effect (CCE) function, which is represented as:

(5) $$y_{it}=\alpha_{1i}+\beta_{i}x_{it}+\delta_{i}{\bar{x}}_{it}+\eta_{i}{\bar{y}}_{it}+\varphi_{i}f_{t}+\varepsilon_{it}$$

where the independent and dependent variables are represented as $x_{it}$ and $y_{it}$, respectively. The slope and the heterogeneous fixed effects of each unit are represented as $\beta_{i}$ and $\alpha_{i}$, respectively. $f_{t}$, $\varphi_{i}$, and $\varepsilon_{it}$ denote the unobserved common effects, the heterogeneous factor loadings, and error term, respectively. Because our desire is in $\beta_{i}$, the estimates of coefficients, Pesaran [83] indicated that the CCEMG estimator is the mean of the CCE estimator ${\overset{⌢}{\beta }}_{CCE,i}$ which can be expressed as:

(6) $${\overset{⌢}{\beta }}_{CCEMG}=N^{-1}\sum_{i=1}^{N}{\overset{⌢}{\beta }}_{CCE,i}$$

where ${\overset{⌢}{\beta }}_{i}$ is the cross-sectional (or individual) coefficient computed from Equation (5).

Eberhardt and Bond [84] created the AMG estimator. Although CCEMG and AMG share similarities, the AMG estimator uses a two-step computation procedure. First, in the first-difference OLS equation, the AMG estimator incorporates (1) the time dummies (D) and (2) the unobserved common factor. This is written as:

(7) $$\Delta y_{it}=\alpha_{1i}+\beta_{i}\Delta x_{it}+\varphi_{i}f_{t}+\sum_{t=1}^{T}\gamma_{t}D_{t}+\varepsilon_{it}$$

where ∆, γ, and *D* are the first difference operator, time dummy, and time dummies, respectively. Second, the AMG calculates the slopes in each unit using the CCEMG mean estimation process stated in Equation (6), which is written as follows:

(8) $$AMG=N^{-1}\sum_{i=1}^{N}{\tilde{\beta }}_{i}$$

where ${\tilde{\beta }}_{i}$ represent the estimates $\beta_{i}$ in Equation (7). Because of their ability to deliver efficient, robust, and accurate results in the presence of slope heterogeneity and cross-sectional dependence, the AMG and CCEMG estimators were chosen for this research [82,84]. Researchers such as Li et al. [39] and Musah et al. [85] validate the applicability of AMG and CCEMG in the presence of cross-sectional dependence, slope heterogeneity, and cointegration in panel data studies. Although both estimators—AMG and CCEMG—are robust to cross-sectional dependency and parameter variability, the AMG estimator is more efficient and unbiased for various time dimensions and cross-sectional combinations [86].

#### 3.4.5. Model Validity Testing

Checking the validity of the model testing is very useful since the heteroscedasticity and serial correlation could lead to inaccurate estimates that could result in biased inferences [77]. Following the literature, we used the Breusch–Pagan/Cook–Weisberg test for heteroscedasticity [87] and the Wooldridge serial correlation test of Wooldridge [88] to test for the validity of the AMG and CCEMG models. The Wooldridge test tested the null hypothesis of no serial correlation in the error terms, whilst the Breusch–Pagan/Cook–Weisberg test tested the null hypothesis of no heteroscedasticity in the residuals.

#### 3.4.6. Granger Causality Test

This study applied Dumitrescu and Hurlin’s [23] (D-H) test for Granger causality in panel data to test for the causalities among the variables. The proposed D–H test is suitable for testing causalities (between explanatory and outcome variables) in panel data using bootstrap options. The test operates under the null hypothesis of the absence of causality. The underlying regression of the D-H panel Granger causality test is written as:

(9) $$y_{i,t}=\alpha_{i}+\sum_{k=1}^{K}\gamma_{ik}y_{i,t-k}+\sum_{k=1}^{K}\beta_{ik}\chi_{i,t-k}+\varepsilon_{i,t}$$

Under the time-invariant assumption, $y_{i,t}$ and $\chi_{i,t}$, are two stationary variables with coefficients that can fluctuate between individual observations. *K* denotes lag order.

## 4. Results and Discussion

### 4.1. Cross-Sectional Dependency and Slope Homogeneity Testing

Table 5 presents the cross-sectional dependency test results of Pesaran [71] and Pesaran [72] for the respective regional economies. CD-test and CD2-test are the results for Pesaran [71] and Pesaran [72], respectively. The alternative hypothesis is that there is a strong cross-sectional dependency among the series. Both results show evidence to support cross-sectional dependency in the MENA region as well as HICs and MICs (see Table 5). In the presence of CD in the panels, it is recommended that the second-generation unit root test, is robust and reliable for checking for a unit root in our study [18].

Likewise, the null hypothesis of slope homogeneity for all the samples is rejected according to the Pesaran–Yamagata [74] test results presented in Table 6. Therefore, heterogeneous panel data models will be appropriate for this study.

### 4.2. Panel Unit Roots Results

The CIPS and CADF results in Table 7 show that some of the variables are significant at a level across the regions while others are stationary at first difference. It implies that some variables are stationary at a level while others are not. While CO_2_ and URB were stationary at a level across all the panels, GEF is stationary at the level only in the HICs. NRE was stationary at a level in the MENA region and HICs. EGR is not stationary at a level across the panels. However, all the variables are stationary at levels. The results show a combination of stationary at level I(0) and, at the first difference, I(1). The unit root results show that the variables exhibit the properties of I(0) and I(1), suggesting that panel econometric models that handle I(0) and I(1) should be employed to analyze the data for this study.

### 4.3. Panel Regression Results

Table 8 presents Westerlund’s [78,80] cointegration results of the relationship among GEF, NRE, URB, EGR, and RE for CN equation for the MENA region as well as HICs and MICs. Based on the statistics value and the *p*-value, the null hypothesis of cointegration is rejected suggesting that cointegration exists across all regional panels. The results in Table 8 further support the appropriateness of using second-generation panel data estimators such as AMG and CCEMG for the analysis.

### 4.4. AMG and CCEMG Results

Table 9 presents the results of the panel AMG and CCEMG for the panels using CO_2_ as an index of CN. It must be emphasized that since CN simply means net-zero emissions, a negative impact of the explanatory variable on CO_2_ implies that the variable promotes the CN agenda while a positive impact implies it depresses the CN agenda. The results in Table 9 show that GEF negatively influenced the CO_2_ of MENA, with a stronger impact on HICs than MICs. The negative impact of GEF on CO_2_ indicates that an effective government is responsible for achieving CN in the MENA. That is, GEF in terms of credibility and formulating quality CN policies, as well as a strong commitment to putting these policies into action, is critical for attaining the CN objective in the MENA region. This is not surprising since MENA governments over the years have taken initiatives, such as the MENA–OECD initiatives, towards effective governance, regulatory, and policy-making capacities [63]. Similarly, MENA’s World Bank roadmap for climate change provides support to mainstream climate action in core government functions and institutions by facilitating sectoral and vertical integration of climate change through well-coordinated government strategies and policies [3]. Generally, this provides evidence to support Khan et al. [10] who revealed that institutional quality promotes CN among G-7 nations.

The results show that NRE hinders CN by increasing CO_2_ in the MENA region for both HICs and MICs. The key sources of CO_2_ in the MENA region are energy production and consumption, such as oil and natural gas [89]. Consistent with most previous studies [90,91,92], our findings indicate that the rich reserves and low cost of NRE have facilitated the development and use of large-scale high-energy equipment and production processes. Higher NRE causes greater emissions in the MENA, as predicted because most of the countries in the region are oil- or gas-reliant, whether they are importers or exporters of such fossil fuels. Our findings also corroborate with Shafiei and Salim [68] and Deng et al. [69] who found energy consumption as a key determinant of CO_2_. Similarly, the findings indicate that EGR postpones CN by increasing CO_2_ in the MENA area. As expected, EGR initially promotes CO_2_, affecting CN negatively in the MENA area, because of the region’s high reliance on NRE for economic development. Thus, initial economic development driven by energy usage adds to pollution in the MENA area. This conclusion backs the findings of Abbasi et al. [24], Shao et al. [8], and Qin et al. [6], who discovered that increased EGR dampens the environment delaying the CN objective owing to natural resource exploitation, which is related with high pollution [44,93]. However, our findings differ from Bekhet et al. [35], who discovered that economic development in the GCC reduces CO_2_.

RE promotes CN by reducing CO_2_ in the MENA region. Similar findings are observed across the panels (HICs and MICs). This is consistent with Khan et al. [10] and Li et al. [39], who found RE promotes CN. Consequently, the MENA region’s CN goal becomes attainable when people transition toward RE sources, such as wind power, solar power, biomass, and hydropower, which emit little, or no, CO_2_ as compared to traditional energy sources that emit a lot of pollution [22]. A paradigm change from NRE to RE technologies, such as renewables, carbon capture, and improved energy efficiency, among other things, might aid in decoupling growth from CO_2_, which is critical for reaching the CN goal [52]. As indicated in the literature, many MENA nations are becoming less carbon-intensive along with economic expansion for multiple reasons. While some countries have attempted to transition their energy systems away from fossil fuels by investing heavily in RE sources, others utilize less energy per unit of economic activity [52,94].

Furthermore, URB hinders the CN objective across all the panels by increasing CO_2_ in the MENA region. The MENA countries are becoming more urbanized [95]. As the MENA region becomes more urbanized, CO_2_ increases, thus prolonging the CN objective [95]. Our findings provide further evidence to support Khan et al. [59] and Zeeshan et al. [67] revealed that URB through anthropogenic activities, such as the transportation, farming, and industrial operations increases CO_2_. The Breusch–Pagan and Wooldridge results presented in Table 9 show no evidence of heteroscedasticity or autocorrelation across the sample, suggesting that the results are valid and reliable.

### 4.5. Results from EKC Test

Table 10 displays the EKC estimates from the AMG and CCEMG estimators. Contrary to the initial EGR-CO_2_ results, the results in Table 10 show an inverted-U shaped relationship between EGR and CO_2_ among MENA nations (at a 10% significant level), particularly among HICs (at a 5% significant level), as shown by significantly positive and negative EGR and EGR2, respectively. This inverted U-shaped relationship between EGR and CO_2_ validates the EKC theory among MENA nations, particularly HICs. In the MENA region, our findings contradict the findings of Al-Rawashdeh et al. [33] who could not establish EKC in the MENA region. It does, however, back up the findings of Sileem [30] and Andriamahery and Qamruzzaman [14], who verified EKC in MENA nations. The evidence for the EKC hypothesis among MENA countries, particularly those in the HICs, suggests that CO_2_ initially increases with a country’s income level until it reaches a certain threshold, after which further increases in income level result in strict environmental regulations, industry standards, and R&D investment, resulting in new, cleaner, and environmentally friendly technologies to replace old and higher-emission technologies. Thus, EGR initially impedes CN due to the scale impact, but this is reversed later due to the composition and technique effect [50,52], resulting in CN. The feasibility of EKC in the MENA area is owing to the region’s attempts to offset the economic impact of climate activities. Climate Action in the MENA (CAMENA), launched in 2014 by the European Investment Bank and the United Kingdom, is one of the region’s most popular programs. CAMENA is a financial institution that promotes sustainable economic and climate-change operations in the MENA area. Its regional strategic targets include environmental and social advantages, increased resilience to climate change, and EGR with decreased greenhouse gas emissions [96].

**Figure 1:**
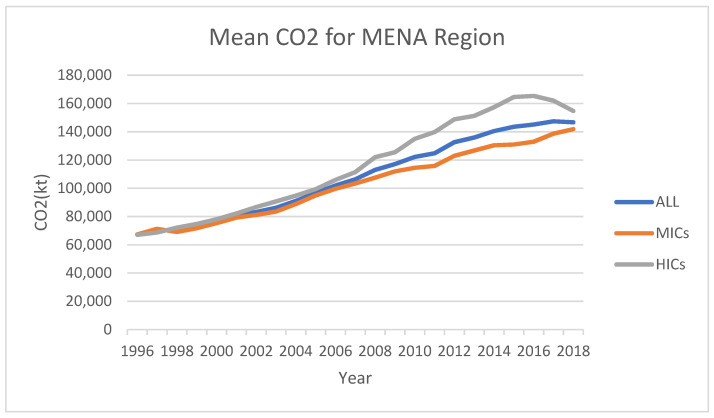
Mean CO_2_ for Mean Region.

An examination of the data reveals that, on average, HICs in the MENA region reached their CO_2_ peak years in 2016 (as shown in Figure 1), with a per capita income approximately USD 25,000.00. Furthermore, the selected MENA countries, as a whole, hit their peak period in 2017 (albeit statistical significance is weak) with an income per capita of approximately USD 16,000.00. However, given the weak responsiveness of CO_2_ to EGR, as shown by the elasticities in Table 10, achieving the net-zero emission target in the MENA region would be challenging. Furthermore, since their peaks up to 2018, CO_2_ levels in HICs and all selected MENA countries have decreased by about 800 kt and 1000 kt, respectively. This finding is consistent with Lamb et al. [97] who found weak evidence for EKC among richer countries.

Even though EGR and EGR2 estimates for MICs in the MENA area are positive and negative, respectively, EGR2 was not statistically significant. This means that, while there was evidence of EKC in the MENA region’s MICs, it was not statistically proven. This conclusion implies that, while MICs in the MENA area have undertaken growth–climate mitigation initiatives, much more has to be done to meaningfully benefit from the programs. This finding validates the findings of Arouri et al. [12], who found weak evidence to justify EKC in MENA nations. Since this study used consumption-based emissions (which continue to rise) as a CN index, in practice, it implies that the peak consumption-based emissions may be much more delayed. In general, our findings agree with those of He et al. [42] and Ntarmah et al. [16], who discovered a mixed outcome of EKC among heterogeneous groups within a geographical region. Despite the similarity of AMG and CCEMG, along with the Breusch–Pagan and Wooldridge figures, no heteroscedasticity or autocorrelation statistics reported in Table 10 show that the results are dependable and genuine.

### 4.6. Granger Causality Results

As indicated earlier, the Dumitrescu and Hurlin [23] Granger causality test was conducted to explore the causalities among the variables. The study explored causalities for the whole sample and the respective regions. Table 11 presents the results of the Granger causality tests.

The causality results in Table 11 showed that there was a feedback mechanism between EGR and CO_2_ across the panels. This means that regardless of being HICs or MICs in the MENA region, EGR influences CO_2_ and vice versa. This finding supports Gorus and Aydin [20] and Mensah et al. [18] who revealed a bidirectional causal relationship between EGR and environmental variables. In contrast, the finding of this study rejects Hu et al. [34] results, which revealed either unidirectional or no causal links between EGR and environmental variables. Similarly, this study revealed bidirectional causal links between URB and CO_2_ across HICs and MICs in the MENA region. That is, as URB increases CO_2_ in the MENA region, CO_2_ also influences urban development in the region. The findings show that feedback effects exist among the variables. The study, therefore, provides support for the studies [71,98,99] that established bidirectional relationships among environmental variables and the determinants. Generally, the causality results presented by this study provide further support for the importance of taking into consideration reverse causality in environmental and macroeconomics studies.

**Figure 2:**
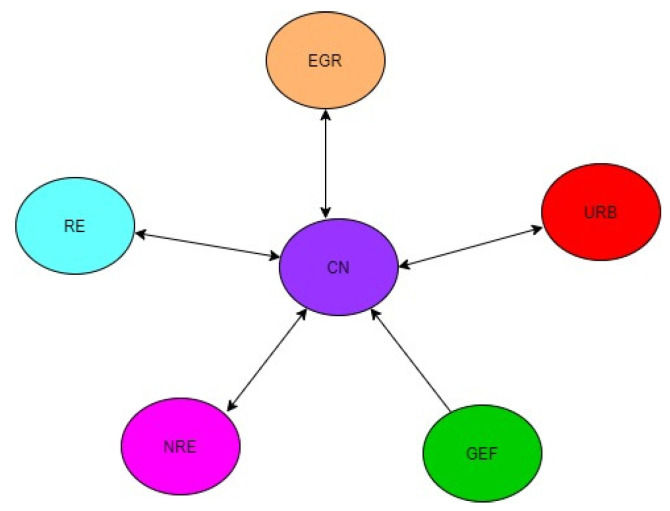
Causalities for all the 16 MENA Countries selected for the Study.

**Figure 3:**
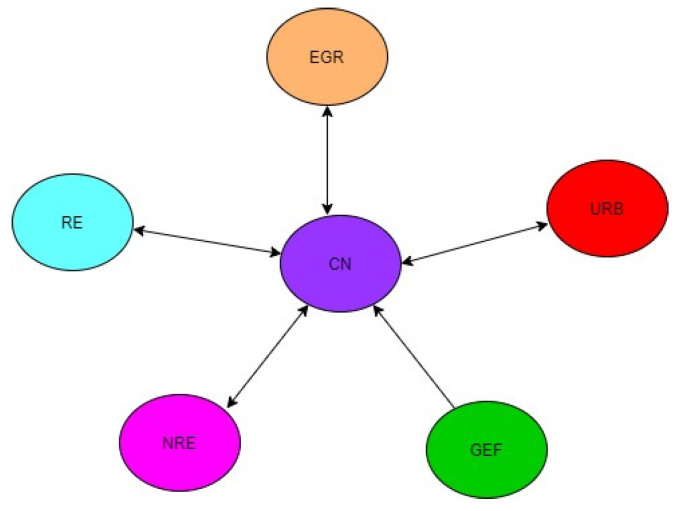
Causalities for the 10 selected MICs in the MENA Region.

**Figure 4:**
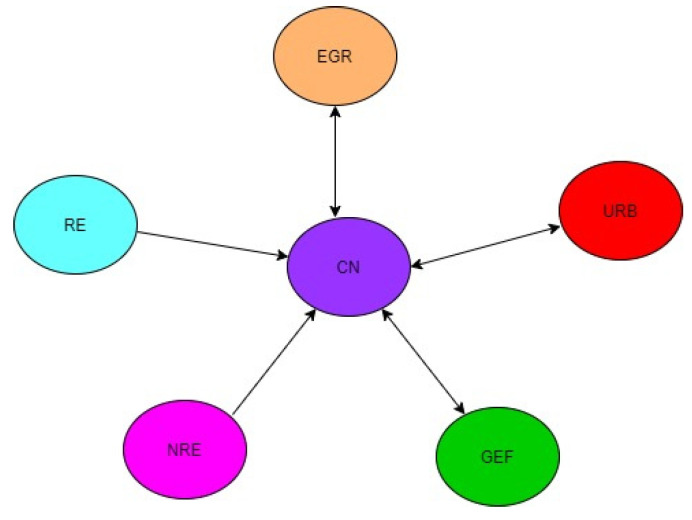
Causalities for HICs in MENA.

While there is a bidirectional causal relationship between NRE, RE, and CO_2_ in the MENA region, precisely regarding MICs, there is a unidirectional causal link from NRE and RE to CO_2_ among HICs in the MENA region. Furthermore, there is a unidirectional causal link from GEF to CO_2_ in the MENA region and MICs but a bidirectional causal link between GEF and CO_2_. These mixed results demonstrate that the causal dynamics between NRE, RE, GEF, and CO_2_ differ across income levels in the MENA region. Thus, the findings clarify the mixed findings reported by many scholars [77,100,101]. This variation in the results across income levels may be due to variation in the level of commitments these groups put towards achieving CN. Since commitments (in terms of policy initiatives, financial and growth opportunities, and alternatives) to CN are not the same for the heterogeneous groups, it is not surprising that someone group (say, HICs) exhibited strong associations in terms of magnitude and elasticities among the variables than others. The results imply that CN through decoupling NRE, URB, and EGR from CO_2_ will have positive repercussions on the economy as a whole. Figure 2, Figure 3 and Figure 4 graphically illustrate the causality results.

## 5. Conclusions

To contribute to developing CN from the theoretical to the practical stage, this study investigated the roles of RE, EGR, and GEF in CN from the perspective of the MENA countries. Using panel data of 16 MENA countries from 1996 to 2018, we applied heterogeneous panel data methodologies and second-generational econometric estimators (AMG and CCEMG) that are robust to cross-sectional dependence and slope heterogeneity. We discovered that MENA data are cross-sectionally dependent, heterogeneous, integrated of order one, I(1), and cointegrated. We observed that the GEF helps to achieve the CN objective in the MENA area, with a larger marginal impact in MICs than in the HICs. Similarly, RE contributes to the CN goal in MENA nations. EGR, on the other hand, initially delays the goal of CN across the HICs and MICs. However, when examined within the EKC framework, we discovered that the detrimental impact of EGR on CN in the MENA area, particularly the HICs, is reversed at a later stage of economic advancement, implying that EGR at an advanced level supports CN when technique effect is in place. Thus, EKC for CO_2_ was established in the MENA area, particularly among the HICs. Although there were signs of MICs, this was not significantly validated. NRE and URB both had a controlling influence on CN, delaying CN in the MENA area. Furthermore, we discovered a feedback mechanism between (i) EGR and CN and (ii) URB and CN in the MENA area, encompassing the sub-regions of the MICs and the HICs, indicating that EGR and URB Granger produce CN and vice versa. While we found a feedback mechanism between NRE and CN in MENA, particularly in the HICs, we only identified a one-way causal relationship between NRE and CN in MICs. Finally, we found a one-way causal link from GEF and RE to CN in the MENA region.

Based on the findings, the following recommendations focus on improving the roles of RE, GEF, and EGR towards CN objectives in the MENA region.

First, policy actions aimed at shifting NRE to RE in the MENA area should be supported. As a result, we urge that authorities in the MENA area increase their investments in RE. This might be an infrastructure investment (i.e., the development of green employment for the clean production process) and the implementation of prizes and incentives to encourage businesses and organizations to move from NRE to RE. Policymakers should create policies that favor the use of solar, wind, and hydropower instead of electricity.

Second, GEF reforms that increase regulatory and policy-making capacities are required to meet the region’s CN objectives. We urge that governments in the MENA area develop a comprehensive grasp of the components of an effective CN policy. Governments must take an active role in developing and implementing successful environmental policies, laws, and designs. Effective governance is required for the implementation of plans and policies to generate the essential investment, innovation, and change. Furthermore, governments and the corporate sector should collaborate to develop realistic carbon neutral policies and devote greater resources to changing technology and identifying innovative ways to reduce CO_2_.

Third, the presence of EKC in MENA, notwithstanding its statistical significance, particularly in MICs, suggests that MENA nations’ CN goals are practicable with consistent efforts and initiatives. As a result, we urge regional governments and policymakers to step up efforts to decouple EGR from CO_2_. Countries with strong RE potential should employ more RE in growth activities, while those with low RE potential should enact modest carbon taxes and other environmental levies, such as “carbon pricing”, to prevent highly polluting firms from delaying the MENA region’s CN. The resulting tax income should be invested in carbon-neutral initiatives. Furthermore, in the face of EKC in the MENA region, we advise these countries to constantly align EGR in order toward net-zero emissions. Greater investment in CN programs and R&D, resulting in new and environmentally benign technologies that replace obsolete and highly polluting technologies, as well as robust environmental laws and industry standards, should be prioritized as income levels rise.

Finally, MENA nations should prioritize the notion of green and sustainable URB to reduce the negative impact of URB on the environment. Furthermore, authorities should work on technical advancements in food and resource production to assist reduce humanity’s overreliance on the natural environment for survival. Furthermore, countries should build rural communities in terms of basic amenities to decrease rural–urban migration.

Regardless of the robustness of our findings, we recognize the following study limitations. First, while generalizations may be taken for other areas, the findings of this study are limited to the MENA nations studied. Second, this study only looked at income groups (HICs and MICs), not sub-regions or specific nations in the region. As a result, policy choices based on the study should consider the MENA nations and income groups as a whole, rather than just sub-regions or individual countries. Finally, this study used consumption-based emissions as the only index of CN. As a result, the findings are applicable to consumption-based emissions. Future research might look at specific countries’ progress toward CN.

## Figures and Tables

**Table 1:** Summary of Environmental Literature.

Authors	Variables	Method	Sample	Time	Key Findings
Bekhet et al. [35]	CO_2_, EGR	DSEM	GCC	1980–2011	Monotonic decreasing
Djellouli et al. [36]	GDP, CO_2_	ARDL	Africa	2000–2015	Monotonic increasing
Esso and Keho [37]	EC, EGR, CO_2_	Cointegration, Causality	12 SSA	1971–2010	mixed across countries
Gyamfi et al. [38]	EC, EGR, CO_2_	ARDL	E-7 nations	1995–2018	Bidirectional
Li et al. [39]	EC, EGR, CO_2_	CCEMG and the AMG	G20 countries	1992–2014	EC and EGR increase CO_2_
Destek and Sinha [40]	EF, EGR, REC	FMOLS, DOLS	OECD	1980–2014	U-shaped
Ntarmah et al. [16]	REC, BF, EGR, CO_2_	Panel VAR	SSA	1990–2018	Multiple- shapes
Ehigiamusoe and Dogan [22]	REC, EGR, CO_2_	AMG, FMOLS, DOLS	LICs	1990–2016	Positive interaction effect
Gorus and Aydin [20]	EC, EGR, CO_2_	Granger causality	MENA	1975–2014	Bidirectional
Halkos and Polemis [41]	CO_2_, EGR	OLS, GMM	OECD	1970–2014	N-shaped
Xue et al. [19]	URB, CO_2_, EGR	ARDL	France	1987–2019	Monotonic increasing
He et al. [42]	EGR, CO_2_, GOV	Spatial Regression	Cities in China	2001–2018	Multiple-shapes
Jebli et al. [43]	CO_2_, EGR, REC	FMOLS, DOLS	OECD	1980–2010	EKC
Mensah et al. [18]	CO_2_, EGR, URB	AMG, CCEMG	SSA	1990–2018	Monotonic increasing Bidirectional
Ntarmah et al. [44]	CS, EGR, CO_2_, POP, REC	Panel VAR	SSA Sub-regions	1990–2018	Multiple-shapes
Zeraibi et al. [45]	GE, CO_2_, EGR	GMM, FMOLS	China	2007–2017	N-Shaped
Rahman and Vu, [17]	URB, EGR, CO_2_, REC	ARDL, VECM	Australia, Canada	1960–2015	Monotonic increasing Bidirectional
Sarkodie et al. [46]	REC, CO_2_, ES	neural network, SIMPLS, ARDL	China	1961–2016	EKC

**Table 2:** Summary of Carbon Neutrality Studies.

Authors	Variables	Method	Sample	Time	Key Findings
Khan et al. [10]	IQ, RE, CE	Panel Data Models	G-7 nations	1990–2018	IQ and RE promotes CE
Hu et al. [34]	EC, EGR, CE	Time series analysis	India	1990–2018	RE delays CE
Abbasi et al. [24]	NRD, EGR, EC, POP, CE	ARDL	UK	1970–2019	EGR, EC, NRD negatively affect CE
Zhang [47]	TI, EGR, CE	STIRPAT model	BRICS	1990–2019	TI promotes CE, EGR discourages CE
Li and Haneklaus [7]	EGR, TO, CE	ARDL	China	1992–2020	EKC, TO influence CE
Shao et al. [8]	ERR&D, RER&D, EGR, CE	DOLS, FMOLS	USA	1990–2019	RER&D and ERR&D positively contribute to CE; EGR discourages CE
Iqbal et al. [48]	EXPD, EI, CE	AMG	OECD	1990–2019	EXPD and EGR damages CE
Koondhar et al. [49]	BioE, CE, ABEG	ARDL	China	171–2019	BioE promotes CE
Qin et al. [6]	FD, RE, EGR, CE	Maki Cointegration	China	1988–2018	FD, RE promotes CE, EGR discourages CE
Chien et al. [9]	EI, Etax, GG, CE	QARDL	USA	1970–2015	EI, Etax, and GG promote CE
Shen et al. [50]	EGR, RE, CE	DOLS, FMOLS	BRICS	1980–2018	EKC, RE promotes CE
Safi et al. [51]	RE, EGR, FDI, CE	Panel Data Models	G-7 nations	1990–2019	Etax, ER&D promotes CE; EGR discourages CE
Udemba and Alola [25]	ECI, RE, CE	Cointegration	Australia	1996 Q1–2018 Q4	RE promotes CE; EGR and FDI hampers CE
Zheng et al. [52]	ECI, RE, CE	AMG, DOLS	16 major export economies	1990–2019	EKC; ECI and RE promote CE
Li et al. [39]	EXPD, TO, RE, EGR, CE	Time series analysis	China	1989–2019	EXPD and RE promote CE; TO and EGR hinders CE

Note: CE (carbon neutrality), CO_2_ (Carbon Dioxide Emissions), ECI (Economic Complexity Index), URB (Urbanization), Etax (Environmental Tax), EGR (Economic Growth), EXPD (Export Diversification), HP (Haze Pollution), TO (Trade Openness), R&D (Research and Development), ERR&D (Environmental research and development), RER&D (renewable energy R&D), EF (Ecological Footprint), CS (Credit Supply), GE (Government Expenditure), BF (Bank Financing), POP (Population), EI (Energy Innovation), RE (Renewable Energy), GOV (Governance), SSA (Sub-Saharan African), OECD (Organization for Economic Co-operation and Development), ES (Environmental Sustainability), LICs (Low-Income Countries), GCC (Gulf Cooperation Council), OLS (Ordinary Least Squares), FMOLS (Fully Modified OLS), ARDL (Autoregressive Distributed Lag), GMM (Generalized Method of Moments), VAR (Vector autoregression), VECM (Vector Error Correction Model), AMG (Augmented Mean Group), CCEMG (Common Correlated Effects Mean Group), DOLS (Dynamic OLS), DSEM (Dynamic Structural Equation Modeling), and BioE (bioenergy).

**Table 3:** Descriptive Statistics and Normality Results.

	OBS	Mean	Std. Dev.	Min	Max	Skewness	Kurtosis	Jarque–Bera	Probability
	MENA								
lnEGR	368	8.932	1.103	3.955	11.039	−0.159	3.268	2.660	0.265
lnCO_2_	368	1.601	1.010	−0.697	3.417	−0.226	2.610	5.461	0.065
lnURB	368	4.315	0.207	3.753	4.605	−1.099	4.049	90.913	0.000
lnNRE	368	7.479	1.063	5.073	9.394	−0.146	2.599	3.769	0.152
lnRE	368	1.527	1.363	0.000	4.221	0.370	1.811	30.083	0.000
lnGEF	368	−0.115	0.664	−1.483	1.233	0.219	2.315	10.125	0.006
	HICs								
lnEGR	138	9.821	1.002	7.413	11.039	−1.366	3.620	45.145	0.000
lnCO_2_	138	2.567	0.558	1.087	3.417	−0.364	2.330	5.643	0.059
lnURB	138	4.465	0.100	4.270	4.605	−0.292	2.077	6.860	0.032
lnNRE	138	8.501	0.607	7.571	9.394	−0.198	1.634	11.788	0.003
lnRE	138	0.573	1.000	0.000	2.921	1.401	3.135	45.241	0.000
lnGEF	138	0.485	0.506	−0.848	1.233	−0.343	2.120	7.160	0.028
	MICs								
lnEGR	230	8.399	0.767	3.955	10.207	−1.011	9.306	420.275	0.000
lnCO_2_	230	1.021	0.741	−0.697	2.239	−0.655	3.162	16.729	0.000
lnURB	230	4.226	0.203	3.753	4.550	−1.001	3.396	39.920	0.000
lnNRE	230	6.865	0.762	5.073	8.144	−0.622	3.288	15.603	0.000
lnRE	230	2.099	1.226	0.059	4.221	0.071	1.864	12.553	0.002
lnGEF	230	−0.474	0.456	−1.483	0.606	−0.046	2.552	2.000	0.368

**Table 4:** Correlation and Multi-collinearity Results.

	lnCO_2_	lnEGR	lnURB	lnNRE	lnRE	lnGEF	Tol	VIF
MENA								
lnCO_2_	1							
lnEGR	0.529	1					2.590	0.386
lnURB	0.408	0.322	1				1.380	0.725
lnNRE	0.491	0.501	0.489	1			2.660	0.376
lnRE	−0.487	0.395	−0.302	−0.584	1		3.240	0.309
lnGEF	−0.419	0.434	0.393	−0.408	0.184	1	1.450	0.690
HICs								
lnCO_2_	1							
lnEGR	0.554	1					3.850	0.260
lnURB	0.328	−0.151	1				1.300	0.769
lnNRE	0.464	0.507	−0.260	1			2.380	0.420
lnRE	−0.568	0.156	0.297	−0.562	1		1.970	0.508
lnGEF	−0.319	−0.231	0.365	−0.428	0.413	1	1.630	0.613
MICs								
lnCO_2_	1							
lnEGR	0.390	1					1.300	0.769
lnURB	0.212	0.210	1				1.050	0.952
lnNRE	0.486	0.367	0.167	1			2.640	0.379
lnRE	−0.425	0.170	−0.368	−0.522	1		2.350	0.426
lnGEF	−0.158	0.187	0.219	−0.146	0.285	1	1.150	0.870

Tol means Tolerance; VIF means Variance Inflation Factor. lnCO_2_ is the dependent variable.

**Table 5:** Pesaran Cross-Sectional Dependency Results by Economic Regions.

	CD-Test			CD2-Test		
	MENA	HICs	MICs	MENA	HICs	MICs
lnGEF	5.257 ^b^	6.968 ^a^	3.095 ^b^	22.108 ^a^	26.264 ^a^	11.694 ^a^
lnNRE	8.784 ^a^	10.068 ^a^	7.600 ^a^	20.434 ^a^	21.219 ^a^	17.566 ^a^
lnCO_2_	5.907 ^b^	9.429 ^a^	6.084 ^a^	18.384 ^a^	20.150 ^a^	11.495 ^a^
lnURB	4.096 ^b^	7.587 ^a^	3.045 ^b^	22.213 ^a^	25.315 ^a^	12.707 ^a^
lnEGR	18.404 ^a^	22.666 ^a^	11.509 ^a^	24.210 ^a^	28.311 ^a^	16.728 ^a^
lnRE	4.944 ^b^	6.805 ^a^	4.318 ^b^	16.289 ^a^	19.253 ^a^	14.607 ^a^

^a,b^ denotes significance at 1% and 5% levels, respectively.

**Table 6:** Pesaran–Yamagata (2008) [74] Slope Homogeneity Results.

Test	MENA	HICs	MICs
∆	7.643 ^a^	8.534 ^a^	4.957 ^a^
∆Adj	10.190 ^a^	12.768 ^a^	6.075 ^a^

^a^ denotes significance at 1% and 5% levels, respectively.

**Table 7:** Panel Unit Roots Results.

	CIPS			CADF		
	MENA	HICs	MICs	MENA	HICs	MICs
Level						
lnGEF	−1.909	−2.744 ^b^	−1.517	−1.849	−2.658 ^b^	−1.469
lnNRE	−3.645 ^a^	−2.955 ^a^	−1.814	−3.312 ^a^	−2.689 ^b^	−1.648
lnCO_2_	−2.656 ^b^	−2.533 ^b^	−2.139 ^b^	−2.400 ^b^	−2.286 ^b^	−1.931
lnURB	−2.269 ^b^	−3.031 ^a^	−3.159 ^a^	−2.238 ^b^	−2.936 ^a^	−3.060 ^a^
lnEGR	−1.290	−1.269	−1.758	−1.249	−1.229	−1.703
lnRE	−1.683	−2.011 ^b^	−1.901	−1.630	−1.948	−1.841
First Difference						
lnGEF	−2.225 ^b^	−2.472 ^b^	−2.563 ^b^	−2.065 ^b^	−2.294 ^b^	−2.378 ^b^
lnNRE	−4.626 ^a^	−4.850 ^a^	−3.604 ^a^	−4.203 ^a^	−4.407 ^a^	−3.275 ^a^
LnCO_2_	−4.647 ^a^	−4.817 ^a^	−3.837 ^a^	−4.195 ^a^	−4.348 ^a^	−3.464 ^a^
lnURB	−3.765 ^a^	−4.663 ^a^	−5.217 ^a^	−3.494 ^a^	−4.327 ^a^	−4.840 ^a^
lnEGR	−3.335 ^a^	−3.419 ^a^	−3.271 ^a^	−3.094 ^a^	−3.173 ^a^	−3.035 ^a^
lnRE	−3.168 ^a^	−4.589 ^a^	−2.589	−2.940 ^a^	−4.258 ^a^	−2.402 ^b^

^a,b^ denotes significant at 1%, 5%, and 10% significance levels, respectively.

**Table 8:** Westerlund [78,80] Cointegration Results.

	Westerlund [78] Results				Westerlund [80] Results		
	MENA	HICs	MICs		MENA	HICs	MICs
Gt	−9.050 ^b^	−8.850 ^b^	−7.953 ^b^	DHg	3.236 ^a^	3.642 ^a^	2.755 ^b^
Pt	−11.751 ^a^	−12.208 ^a^	−12.800 ^a^	DHp	3.560 ^a^	3.976 ^a^	3.014 ^a^
Ga	−13.691 ^a^	−14.251 ^a^	−13.585 ^a^				
Pa	−13.229 ^a^	−15.111 ^a^	−14.534 ^a^				

^a,b^ denotes significance at 1% and 5% levels, respectively. The bootstrap method was used. Gt, Ga, Pt, and Pa are Westerlund [78] results while DHg and DHp are Westerlund [80] results.

**Table 9:** AMG AND CCEMG Results.

	MENA		HICs		MICs	
	AMG	CCEMG	AMG	CCEMG	AMG	CCEMG
lnGEF	−0.394 ^b^	−0.416 ^a^	−0.231 ^b^	−0.237 ^a^	−0.525 ^a^	−0.559 ^a^
lnNRE	0.557 ^a^	0.554 ^a^	0.630 ^a^	0.620 ^a^	0.320 ^a^	0.302 ^a^
lnURB	0.308 ^a^	0.304 ^a^	0.504 ^a^	0.502 ^a^	0.290 ^a^	0.274 ^a^
lnEGR	0.744 ^a^	0.732 ^a^	0.592 ^a^	0.602 ^a^	0.549 ^a^	0.521 ^a^
lnRE	−0.381 ^b^	−0.382 ^b^	−0.502 ^a^	−0.498 ^a^	−0.358 ^b^	−0.336 ^b^
BP Test	0.62 (0.431)		1.55 (0.213)		2.49 (0.115)	
WR Test	1.947 (0.183)		3.305 (0.129)		1.116 (0.318)	

^a,b^ denotes significant at 1%, 5%, and 10% significance levels, respectively. BP and WR represent the Breusch–Pagan test for heteroscedasticity and the Wooldridge test for autocorrelation. *p* values are in parenthesis ().

**Table 10:** EKC Results.

	MENA		HICs		MICs	
	AMG	CCEMG	AMG	CCEMG	AMG	CCEMG
lnGEF	−0.346 ^b^	−0.365 ^b^	−0.203 ^b^	−0.208 ^b^	−0.461 ^a^	−0.493 ^a^
lnNRE	0.489 ^a^	0.486 ^a^	0.553 ^a^	0.544 ^a^	0.281 ^b^	0.265 ^b^
lnURB	0.270 ^b^	0.267 ^b^	0.442 ^a^	0.441 ^a^	0.255 ^b^	0.240 ^b^
lnEGR	0.591 ^a^	0.582 ^a^	0.471 ^a^	0.478 ^a^	0.436 ^a^	0.414 ^a^
lnEGR2	−0.201 ^c^	−0.195 ^c^	−0.262 ^b^	−0.265 ^b^	−0.040	−0.025
lnRE	−0.334 ^b^	−0.335 ^b^	−0.441 ^a^	−0.437 ^a^	−0.314 ^b^	−0.295 ^b^
BP Test	0.67 (0.415)		1.85 (0.174)		3.08 (0.079)	
WR Test	2.321 (0.148)		74.209 (0.119)		1.542 (0.246)	

^a,b,c^ denotes significant at 1%, 5%, and 10% significance levels, respectively. BP and WR represent the Breusch–Pagan test for heteroscedasticity and the Wooldridge test for autocorrelation. *p* values are in parenthesis ().

**Table 11:** Dumitrescu and Hurlin [23] Granger Causality Results.

	MENA	HICs	MICs
lnNRE → lnCO_2_	5.887 ^a^	7.807 ^a^	3.613 ^a^
LnCO_2_ → lnNRE	4.967 ^a^	1.553	6.811 ^a^
lnGEF → lnCO_2_	10.217 ^a^	15.681 ^a^	5.434 ^a^
LnCO_2_ → lnGEF	1.961 ^c^	2.311 ^b^	1.619
lnRE → lnCO_2_	4.105 ^a^	3.153 ^a^	6.935 ^a^
LnCO_2_ → lnRE	6.052 ^a^	1.080	6.293 ^a^
lnURB → lnCO_2_	4.473 ^a^	4.388 ^a^	3.372 ^a^
LnCO_2_ → lnURB	3.118 ^a^	2.720 ^b^	4.603 ^a^
lnEGR → lnCO_2_	6.068 ^a^	7.713 ^a^	4.297 ^a^
lnCO_2_ → lnEGR	8.144 ^a^	10.271 ^a^	5.416 ^a^

^a,b,c^ denotes significance at 1%, 5%, and 10% levels, respectively.

## Data Availability

The datasets generated and/or analyzed during the current study are available in the World Development Indicators repository, https://data.worldbank.org/ (accessed on 16 January 2022), and World Governance Index repository, http://info.worldbank.org/governance/wgi/ (accessed on 16 January 2022).
